# Outcomes Among Patients Hospitalized for COVID-19 Treated with Remdesivir in an Urban Center Pre-COVID-19 Vaccination

**DOI:** 10.1007/s40615-023-01861-6

**Published:** 2023-11-27

**Authors:** Debra Chew, Stephanie Shiau, Sree Sudharshan, Aparna Alankar, Malithi Desilva, Swetha Kodali, Tricia Mae Raquepo, Naema Meilad, Alexander Sudyn, Shobha Swaminathan

**Affiliations:** 1 Division of Infectious Diseases, Rutgers New Jersey Medical School, 185 South Orange Avenue, MSB I-689, Newark, NJ 07101 USA; 2 Department of Biostatistics and Epidemiology, Rutgers School of Public Health, Piscataway, NJ USA

**Keywords:** COVID-19, Remdesivir, COVID-19 Outcomes, Minority Populations, I (health, education, and welfare)

## Abstract

**Objective:**

Data on treatment outcomes among minority populations treated with remdesivir are limited. We sought to evaluate outcomes among patients hospitalized with COVID-19 and treated with remdesivir among a predominantly Black and LatinX population.

**Methods:**

This was a retrospective cohort study of adult patients hospitalized with COVID-19 and treated with remdesivir at an urban hospital in Newark, NJ, between May 1, 2020, and April 30, 2021, prior to widespread COVID-19 vaccination uptake. We describe 28-day mortality by demographic, socio-economic, and clinical factors, including clinical status by World Health Organization’s (WHO) 8-point Ordinal Scale for Clinical Improvement.

**Results:**

A total of 206 patients met study inclusion criteria (52% were male, 41% non-Hispanic Black and 42% Hispanic). Overall mortality at 28 days was 11%. Eighty-one percent of patients with baseline WHO status of 4 or greater recovered by day 14. Mortality was higher among those who were older (*p* = 0.01), those with underlying diabetes mellitus (*p* = 0.047), those with more severe illness on admission by WHO Ordinal Scale (WHO status ≥ 4), and those on concomitant tociluzimab or convalescent plasma use.

**Conclusions:**

We found that remdesivir was effective in treating most COVID-19 patients in our study. Traditional risk factors, such as advanced age and underlying co-morbidities, were associated with worse clinical outcomes and deaths.

## Introduction

Clinical outcomes among patients hospitalized for COVID-19 have been influenced by various factors, including underlying co-morbidities, age, sex, socio-economic factors, laboratory abnormalities, treatment, pathogenicity of the virus strain, and COVID-19 vaccination status [1–14]. The use of SARS-CoV-2 antivirals, such as remdesivir (RDV), has been shown to provide clinical benefit in shortening the time to clinical recovery among patients hospitalized with COVID-19 [15–17]. RDV works by inhibiting the viral RNA-dependent, RNA polymerase, thereby preventing the virus from replication [18]. It was granted an Emergency Use Authorization (EUA) by the Food and Drug Administration (FDA) in May 2020 for the treatment of COVID-19 in hospitalized patients [19]. Currently, COVID-19 Treatment Guidelines by the National Institutes of Health [20] recommends the use of RDV in hospitalized adults with COVID-19 who require oxygen supplementation or who are at high risk of progressing to severe COVID-19. The use of RDV in combination with other treatments, such as dexamethasone, has also been shown to improve outcomes in hospitalized patients with COVID-19 [20].

The COVID-19 pandemic has disproportionately impacted certain racial, ethnic, and socioeconomic groups, and has highlighted disparities in healthcare, housing, and other social determinants of health [10–14]. This disparity was particularly evident early in the COVID-19 pandemic, driven by social determinants of health and barriers to care, such as lack of insurance, transportation, childcare, and occupational and housing conditions, as well as a higher prevalence of underlying medical co-morbidities among racial and ethnic minorities, ultimately leading to a higher risk of exposure to SARS-CoV2 and severe COVID-19 disease [21–23]. However, there is lack of research on the specific socio-economic factors associated with COVID-19 patients treated with RDV.

Given limited real-world data on socio-economic factors and RDV use, we evaluated clinical outcomes at our institution during the pre-vaccination period of the COVID-19 pandemic (primarily delta variant predominance), in Newark, New Jersey (NJ), serving a predominantly Black and Latino population, designated with a high CDC Social Vulnerability index (0.95) and COVID-19 Community Vulnerability Index (0.91) [24].

## Methods

We conducted a retrospective study of adult patients (18 years and older) hospitalized with COVID-19 and who received at least one dose of RDV at University Hospital in Newark, NJ, between May 1, 2020, and April 30, 2021. Patients were excluded they received RDV prior to EUA on May 1, 2020, if their creatinine clearance was less than 30 mL/min, or if they had liver function tests over 10 times the upper limit of normal prior to FDA approval of RDV, and if they had prior hypersensitivity to RDV or any of its components. The study protocol was approved by the Institutional Review Board at Rutgers University.

Data were extracted from electronic medical records for patient demographics, comorbidities, disease severity, disease course, RDV administration, concomitant COVID-19 treatments, adverse events, and clinical outcomes. Social deprivation index (SDI) at the zip code level was obtained from the American Community Survey (ACS) [25]. The SDI is a composite measure of area level deprivation based on seven demographic characteristics collected in the ACS and used to quantify the socio-economic variation in health outcomes.

Disease severity and clinical outcomes were assessed on admission and hospital days 5, 7, 14, 28, or discharge (whichever came first), and were assessed according to the 8-point World Health Organization (WHO) Ordinal Scale for Clinical Improvement [26]. For each assessment, the highest category met on that day was recorded.

The primary outcome was mortality by day 28 or discharge, whichever came first. Secondary outcome was the proportion of patients who had recovered by day 14, defined as a score of 1, 2, or 3 on the ordinal scale.

Patient demographic and clinical characteristics were descriptively summarized using frequencies and proportions, or medians and interquartile ranges, as appropriate. Wilcoxon rank-sum tests (continuous variables) or Chi-square and Fisher’s exact tests (categorical variables) were used to compare demographic, socio-economic, and clinical factors by baseline WHO status and mortality by day 28. Among those with baseline WHO status 4 or greater, we compared demographic, socio-economic, and clinical factors among those who did and did not recover by day 14. All statistical analyses were performed using SAS version 9.4 (Cary, NC, USA).

## Results

A total of 612 patients were hospitalized with COVID-19 and received 1 or more doses of RDV between May 1, 2020, and April 30, 2021. Of these, a convenience sample of 244 patients was randomly selected for chart review. Of those reviewed, 38 were excluded (29 had GFR < 30 ml/min, 3 had liver function tests > 10 times the upper limit of normal, 5 for positive SARS-Co-V-2 PCR outside of study period, and 1 patient with documented hypersensitivity to RDV component). The remaining 206 patients’ charts were included in the analysis.

Baseline demographic and clinical characteristics of the study population are presented in Table 1, stratified by baseline WHO status of 3 or less versus WHO status 4 or greater. The median age of patients was 59 years and ranged from 19 to 96 years. Overall, 41% were non-Hispanic Black (NHB) and 42% were Hispanic. Approximately half (48%) were identified as female. Twenty-six percent were uninsured, 20% were Medicaid-insured, and 25% were Medicare-insured. The median SDI was 98. Prevalent baseline medical co-morbidities included hypertension (52%), diabetes (37%), hyperlipidemia (22%), asthma or COPD (20%), and coronary artery disease (10%). Many patients were overweight (30%) or obese (46%), with a median BMI of 29. A majority of the cohort (62%) had two or more underlying comorbid conditions.

**Table 1:** Patient demographics and clinical characteristics, by baseline WHO status

Characteristic	All patients (*N* = 206)	Baseline WHO status 3 or less (*N* = 61)	Baseline WHO status 4 or greater (*N* = 145)	*p* value
Sex, *N* (%)				
Female	99 (48.1)	27 (44.3)	72 (49.7)	0.48
Male	107 (51.9)	34 (55.7)	73 (50.3)	
Age, median (IQR)	59 (45, 68)	60 (50, 72)	58 (43, 66)	0.16
Age, *N* (%)				
18–49	58 (28.2)	15 (24.6)	43 (29.7)	0.76
50–64	78 (37.9)	24 (39.3)	54 (37.2)	
65 +	70 (34.0)	22 (36.1)	48 (33.1)	
Race/ethnicity, *N* (%)*				
Non-Hispanic White	13 (6.3)	3 (4.9)	10 (6.9)	0.46
Non-Hispanic Black	85 (41.3)	31 (50.8)	54 (37.2)	
Hispanic	86 (41.8)	22 (36.1)	64 (44.1)	
Other non-Hispanic	19 (9.2)	4 (6.6)	15 (10.3)	
Unknown (*3 missing values)	3 (1.5)	1 (1.6)	2 (1.4)	
Insurance status, *N* (%)*				
Medicaid	41 (19.9)	14 (23.0)	27 (18.6)	0.38
Medicare	52 (25.2)	16 (26.2)	36 (24.8)	
Commercial	57 (27.7)	20 (32.8)	37 (25.5)	
Uninsured	53 (25.7)	10 (16.4)	43 (29.7)	
Other/unknown	3 (1.5)	1 (1.6)	2 (1.4)	
Social deprivation index, median (IQR)	98 (95, 100)	98 (94, 99)	98 (95, 100)	0.91
Baseline hypertension, *N* (%)	108 (52.4)	31 (50.8)	77 (53.1)	0.76
Baseline diabetes, *N* (%)	77 (37.4)	24 (39.4)	53 (36.6)	0.83
Baseline hyperlipidemia, *N* (%)	45 (21.8)	18 (29.5)	27 (18.6)	0.13
Baseline asthma or COPD, *N* (%)	42 (20.4)	12 (19.7)	30 (20.7)	1.0
Baseline coronary artery disease, *N* (%)	21 (10.2)	8 (13.1)	13 (9.0)	0.50
Baseline congestive heart failure, *N* (%)	16 (7.8)	5 (8.2)	11 (7.6)	1.0
Baseline cirrhosis, *N* (%)	10 (4.9)	5 (8.2)	5 (3.5)	0.24
Cancer diagnosis within the past year (excluding skin malignancy), *N* (%)	12 (5.8)	7 (11.5)	5 (3.5)	0.045
Baseline HIV positive status, *N* (%)	4 (1.9)	2 (3.3)	2 (1.4)	0.53
Other immunosuppression diseases, *N* (%)	10 (4.9)	5 (8.2)	5 (3.5)	0.26
Obesity, *N* (%)	94 (45.6)	27 (44.3)	67 (46.2)	0.88
BMI, median (IQR)	29.2 (25.1, 35.5)	28.7 (23.8, 34.9)	29.5 (25.7, 36.5)	0.28
≥ 2 underlying comorbid conditions**	127 (61.7)	40 (65.6)	87 (60.0)	0.45
WHO Ordinal Scale Score on Hospital Day 1, *N* (%)		–	–	
(3) Hospital, no O_2_	61 (23.1)			
(4) O_2_ by mask/nasal cannula	105 (51.0)			
(5) Non-invasive mechanical ventilation (NIV) or high flow O_2_	33 (16.0)			
(6) Invasive mechanical ventilation (IMV)	5 (2.4)			
(7) IMV + Pressors/Renal replacement therapy (RRT)/Extracorporeal membrane oxygenation (ECMO)	2 (1.0)			
Systemic steroids, *N* (%)	176 (85.4)	44 (72.1)	132 (91.0)	0.0009
Tociluzimab use, *N* (%)	12 (5.8)	1 (1.6)	11 (7.6)	0.11
Convalescent plasma use, *N* %)	14 (6.8)	5 (8.2)	9 (6.2)	0.56
Remdesivir duration (days), median (IQR)	4 (3, 4)	4 (3, 4)	4 (3, 4)	0.66
Remdesivir duration ≥ 5 days	16 (7.8)	4 (6.6)	12 (8.3)	0.78
Remdesivir duration ≥ 3 days	176 (85.4)	52 (85.3)	124 (85.5)	1.0
Time from symptom onset to Remdesivir administration (days)	6 (3, 8)	7 (4, 8)	6 (3, 8)	0.55

^**^2 or more of the following conditions: hypertension, diabetes, hyperlipidemia, asthma or chronic obstructive pulmonary disease (COPD), coronary artery disease, congestive heart failure, cirrhosis, cancer diagnosis within past year, HIV, other immunosuppression diseases, obesity

Seventy percent of the study population met criteria of severe COVID-19 on admission by baseline O_2_ saturation, with 51% requiring oxygen support by mask or nasal cannula (meeting WHO ordinal scale criteria 4), 16% requiring noninvasive ventilation or high flow oxygen (meeting WHO ordinal scale criteria 5), and 3% required ventilatory support (meeting WHO ordinal scale criteria 6 and 7). Overall, 13% of patients were admitted to the ICU on admission. Of the 206 patients, (85%) received 3 or more days of RDV. Eighty-five percent of patients received systemic steroids. All patients except one were not fully vaccinated at time of their COVID-19 diagnosis.

Overall mortality at 28 days was 11% (23/206). Mortality was higher among those who were older (*p* = 0.01), those with underlying diabetes mellitus (*p* = 0.047), those with more severe illness on admission by WHO Ordinal Scale (WHO status 4 or more), and those on concomitant tociluzimab or convalescent plasma use (Table 2). Among 145 patients with baseline WHO status of 4 or greater, 35% recovered by day 5, 55% by day 7, 81% by day 14, and 86% by day 28. Fifteen percent did not recover by 28 days. Younger patients were more likely to recover by day 14 compared to older patients, though this was not statistically significant. Recovery by day 14 was inversely associated with older age and concomitant tociluzimab and convalescent plasma use (*P* < 0.001 and *p* = 0.001 respectively). Those with underlying diabetes and with HIV at baseline were less likely to recover by 14 days (Table 3).

**Table 2:** Patient demographics and clinical characteristics, by mortality status by day 28

Characteristic	No mortality by day 28 (*N* = 182)	Mortality by day 28 (*N* = 23)	*p* value
Sex, *N* (%)			
Female	89 (48.9)	9 (39.1)	0.38
Male	93 (51.1)	14 (60.9)	
Age, median (IQR)	58 (45, 7)	65 (57, 76)	0.01
Age, *N* (%)			
18–49	53 (29.1)	4 (17.4)	0.06
50–64	72 (39.6)	6 (26.1)	
65 +	57 (31.3)	13 (56.5)	
Race/ethnicity, *N* (%)*			
Non-Hispanic White	9 (5.0)	4 (17.4)	0.12
Non-Hispanic Black	75 (41.2)	10 (43.5)	
Hispanic	76 (41.8)	9 (39.1)	
Other non-Hispanic	19 (10.4)	0 (0.0)	
Unknown (*3 missing values)	3 (1.7)	0 (0.0)	
Insurance status, *N* (%)*			
Medicaid	37 (20.3)	3 (13.0)	0.19
Medicare	41 (22.5)	11 (47.8)	
Commercial	52 (28.6)	5 (21.7)	
Uninsured	49 (26.9)	4 (17.4)	
Other/unknown	3 (1.7)	0 (0)	
Social deprivation index, median (IQR)	98 (95, 100)	95 (87, 99)	0.23
Baseline hypertension, *N* (%)	95 (52.2)	13 (56.5)	0.70
Baseline diabetes, *N* (%)	63 (34.6)	14 (60.9)	0.047
Baseline hyperlipidemia, *N* (%)	41 (22.5)	4 (17.4)	0.87
Baseline asthma or COPD, N (%)	39 (21.4)	3 (13.0)	0.55
Baseline coronary artery disease, *N* (%)	19 (10.4)	2 (8.7)	1.0
Baseline congestive heart failure, *N* (%)	15 (8.2)	1 (4.4)	1.0
Baseline cirrhosis, *N* (%)	9 (5.0)	1 (4.4)	1.0
Cancer diagnosis within the past year (excluding skin malignancy), *N* (%)	10 (5.5)	2 (8.7)	0.67
Baseline HIV positive status, *N* (%)	3 (1.7)	1 (4.4)	0.31
Other immunosuppression diseases, *N* (%)	7 (3.9)	3 (13.0)	0.19
Obesity, *N* (%)	82 (45.1)	11 (47.8)	0.80
BMI, median (IQR)	29.2 (25.1, 35.3)	27.4 (24.5, 42.2)	0.58
≥ 2 Underlying comorbid conditions**	113 (62.1)	14 (60.9)	1.0
WHO ordinal scale score on hospital day 1, *N* (%)			
(3) Hospital, no O_2_	56 (30.8)	5 (21.7)	< .001
(4) O_2_ by mask/nasal cannula	99 (54.4)	6 (26.1)	
(5) Noninvasive mechanical ventilation (NIV) or high flow O_2_	24 (13.2)	8 (34.8)	
(6) Invasive mechanical ventilation (IMV)	2 (1.1)	3 (13.0)	
(7) IMV + Pressors/Renal replacement therapy (RRT)/Extracorporeal membrane oxygenation (ECMO)	1 (0.6)	1 (4.4)	
Systemic steroids, *N* (%)	155 (85.2)	20 (87.0)	1.0
Tociluzimab use, *N* (%)	6 (3.30)	5 (21.7)	0.003
Convalescent plasma use, *N* %)	10 (5.5)	4 (17.4)	0.06
Remdesivir duration (days), median (IQR)	4 (3, 4)	4 (2, 4)	0.13
Remdesivir duration ≥ 5 days	14 (7.7)	2 (8.7)	0.70
Remdesivir duration ≥ 3 days	159 (87.4)	17 (73.9)	0.11
Time from symptom onset to Remdesivir administration (days)	6 (3, 8)	7 (3, 8)	0.72

^**^2 or more of the following conditions: hypertension, diabetes, hyperlipidemia, asthma or chronic obstructive pulmonary disease (COPD), coronary artery disease, congestive heart failure, cirrhosis, cancer diagnosis within past year, HIV, other immunosuppression diseases, obesity

**Table 3:** Patient demographics and clinical characteristics of those with WHO status baseline 4 or greater, by recovery status

Characteristic	Baseline ≥ 4 (*N* = 145)	Did not recover by 14 days (*N* = 28)	Did recover by 14 days (*N* = 117)	*p* value
Sex, *N* (%)				
Female	72 (49.7)	12 (42.9)	60 (51.3)	0.42
Male	73 (50.3)	16 (57.1)	57 (48.7)	
Age, median (IQR)	58 (43, 66)	62 (51.5, 70)	58 (43, 65)	0.12
Age, *N* (%)				
18–49	43 (29.7)	6 (21.4)	37 (31.6)	0.40
50–64	54 (37.2)	10 (35.7)	44 (37.6)	
65 +	48 (33.1)	12 (42.9)	36 (30.8)	
Race/ethnicity, *N* (%)*				
Non-Hispanic White	10 (6.9)	4 (14.3)	6 (5.1)	0.22
Non-Hispanic Black	54 (37.2)	13 (46.4)	41 (35.0)	
Hispanic	64 (44.1)	10 (35.7)	54 (46.2)	
Other non-Hispanic	15 (10.3)	1 (3.6)	14 (12.0)	
Unknown (*3 missing values)	2 (1.4)	0 (0.0)	2 (1.7)	
Insurance status, *N* (%)*				
Medicaid	27 (18.6)	5 (17.9)	22 (18.8)	0.14
Medicare	36 (24.8)	12 (42.9)	24 (20.5)	
Commercial	37 (25.5)	6 (21.4)	31 (26.5)	
Uninsured	43 (29.7)	5 (17.9)	38 (32.5)	
Other/unknown	2 (1.4)	0 (0.0)	2 (1.7)	
Social deprivation index, median (IQR)	98 (95, 100)	96.5 (94, 99)	98 (95, 100)	0.29
Baseline hypertension, *N* (%)	77 (53.1)	15 (53.6)	62 (53.0)	0.96
Baseline diabetes, *N* (%)	53 (36.6)	15 (55.6)	38 (32.5)	0.09
Baseline hyperlipidemia, *N* (%)	27 (18.6)	6 (21.4)	21 (18.0)	0.82
Baseline asthma or COPD,* N* (%)	30 (20.7)	6 (21.4)	24 (20.5)	1.0
Baseline coronary artery disease, *N* (%)	13 (9.0)	2 (7.1)	11 (9.4)	1.0
Baseline congestive heart failure, *N* (%)	11 (7.6)	1 (3.6)	10 (8.6)	0.80
Baseline cirrhosis, *N* (%)	5 (3.5)	1 (3.6)	4 (3.4)	1.0
Cancer diagnosis within the past year (excluding skin malignancy), *N* (%)	5 (3.5)	0 (0.0)	5 (4.3)	0.66
Baseline HIV positive status, *N* (%)	2 (1.4)	2 (7.1)	0 (0.0)	0.02
Other immunosuppression diseases, *N* (%)	5 (3.5)	2 (7.1)	3 (2.6)	0.39
Obesity, *N* (%)	67 (46.2)	16 (57.1)	51 (43.6)	0.20
BMI, median (IQR)	29.5 (25.7, 36.5)	31.1 (26.3, 41.4)	29.2 (25.1, 35.5)	0.17
≥ 2 underlying comorbid conditions**	87 (60.0)	17 (60.7)	70 (59.8)	0.93
Systemic steroids, *N* (%)	132 (91.0)	26 (92.9)	106 (90.6)	1.0
Tociluzimab use, *N* (%)	11 (7.6)	9 (32.1)	2 (1.7)	< 0.001
Convalescent Plasma Use, N %)	9 (6.2)	6 (21.4)	3 (2.6)	0.002
Remdesivir duration (days), Median (IQR)	4 (3, 4)	4 (3, 4)	4 (4, 4)	0.16
Remdesivir duration ≥ 5 days	12 (8.3)	3 (10.7)	9 (7.6)	0.70
Remdesivir duration ≥ 3 days	124 (85.5)	22 (78.6)	102 (87.2)	0.24
Time from symptom onset to Remdesivir administration (days)	6 (3, 8)	6 (3, 8)	6 (3, 9)	0.50

^*^2 or more of the following conditions: hypertension, diabetes, hyperlipidemia, asthma or chronic obstructive pulmonary disease (COPD), coronary artery disease, congestive heart failure, cirrhosis, cancer diagnosis within past year, HIV, other immunosuppression diseases, obesity

RDV was generally tolerated. Only 5 patients had severe adverse events related to RDV. These included 2 with elevated liver function tests greater than 5 times the upper limit of normal, 2 with bradycardia and 1 related to phlebitis at the site of the intravenous line.

## Discussion

In conclusion, our study provides valuable insights into the clinical outcomes of remdesivir treatment among patients hospitalized with COVID-19, particularly in a predominantly Black and Latino patient population with high social and community vulnerability indices (Social Vulnerability Index 0.95 and COVID-19 Community Vulnerability Index 0.91). Despite these challenging patient demographics, we observed that the overall mortality at 28 days in our cohort was comparable to previously reported randomized clinical trials with RDV and a large survey of hospitalized COVID-19 patients [15, 16]. In a survey with over 16,000 patients hospitalized for COVID-19 between March and December 2020, the overall mortality rate was 11%, ranging monthly from 7 to 17% [9]. Compared to death rates during the initial phase of the COVID-19 pandemic, COVID-19 mortality rates among hospitalized patients declined over the course of the pandemic, even before the introduction of wide-spread COVID-19 vaccination (which occurred after our study period). This decrease in mortality rates may be attributed to several factors, including improved clinical management of COVID-19, the development of new therapies, increased access to early treatment, and better resource allocation in institutional systems that were not overburdened.

The majority of patients (81%) who received RDV recovered within 7 days of RDV use, comparable to previously reported findings from RCTs. Several RCTs evaluating RDV use in hospitalized adult patients with COVID-19 showed RDV benefit in shortening time to clinical recovery (10 versus 15 days) [15, 16] or showed improved clinical status compared to standard of care [17]. However, RDV had no effect on 28-day mortality rates in these trials [15–17, 27].

We found that traditional risk factors, such as advanced age and multiple co-morbidities, particularly diabetes, was associated with increased risk for severe COVID-19 and death [1–9] as well as increased time to recovery. Data suggest that co-existence of COVID-19 with comorbidities, such as diabetes, cardiovascular disease, cancer, and others may lead to chronic inflammation, impaired immune response, and reduced host tolerance to organ injury, that may predispose to severe, potentially fatal COVID-19 [28, 29]. Worse outcomes associated with concomitant tocilizumab and convalescent plasma use likely reflected underlying severity of COVID presentation at baseline.

However, we acknowledge the limitations of our study, including its retrospective design and the absence of a control group, which may have influenced the interpretation of our results. As a result, the analyses were primarily descriptive, and the conclusions should be interpreted with caution considering these inherent limitations. Our study also highlights the importance of considering potential disparities in healthcare outcomes, as the limited number of non-White participants limited our capacity to fully assess the impact of race and other socio-economic factors on treatment outcomes.

Regarding safety, RDV was in general well-tolerated in our study, with few adverse events noted. Rare adverse events included abnormal liver function tests, and one case each of bradycardia, and peripheral line-associated phlebitis. These findings are consistent with previous safety assessments and provide reassurance about RDV’s acceptability profile.

## Conclusion

In conclusion, our study provides valuable real-world evidence of RDV's outcomes among a vulnerable patient population, shedding light on its effectiveness in the context of socio-economically disadvantaged minorities. While our findings contribute to the understanding of RDV's clinical performance, further research with larger and more diverse patient cohorts, including controlled prospective studies, is essential to strengthen the evidence base and facilitate evidence-based decision-making in the ongoing battle against COVID-19. Additionally, efforts to address healthcare disparities and promote inclusive research practices will be critical in achieving better health outcomes for all patients affected by this devastating disease.

## Data Availability

The data supporting this study are available by the corresponding author upon reasonable request.
